# Experimental methods of post-growth tuning of the excitonic fine structure splitting in semiconductor quantum dots

**DOI:** 10.1186/1556-276X-7-336

**Published:** 2012-06-22

**Authors:** Johannes D Plumhof, Rinaldo Trotta, Armando Rastelli, Oliver G Schmidt

**Affiliations:** 1 Institute for Integrative Nanosciences, IFW, Dresden, Helmholtzstr. 20, D-01069 Dresden, Germany

**Keywords:** Semiconductor quantum dots, Excitonic fine structure splitting, Anticrossing, Entangled photon pairs

## Abstract

Deterministic sources of polarization entangled photon pairs on demand are considered as important building blocks for quantum communication technology. It has been demonstrated that semiconductor quantum dots (QDs), which exhibit a sufficiently small excitonic fine structure splitting (FSS) can be used as triggered, on-chip sources of polarization entangled photon pairs. As-grown QDs usually do not have the required values of the FSS, making the availability of post-growth tuning techniques highly desired. This article reviews the effect of different post-growth treatments and external fields on the FSS such as thermal annealing, magnetic fields, the optical Stark effect, electric fields, and anisotropic stress. As a consequence of the tuning of the FSS, for some tuning techniques a rotation of the polarization of the emitted light is observed. The joint modification of polarization orientation and FSS can be described by an anticrossing of the bright excitonic states.

## Review

Semiconductor quantum dots (QDs) obtained by epitaxial growth are attracting much interest because of their potential use as building blocks for quantum information processing and communication devices [1]. QDs confine the motion of charge carriers in three-dimensions and are often referred to as artificial atoms. The discreteness of the states in QDs leads together with the Pauli exclusion principle to a maximum occupancy of two electrons/holes per quantum state. This renders QDs ideal sources of single photons, where only one electron-hole pair (which forms a neutral exciton, X, because of the Coulomb attraction), can recombine within the excitonic lifetime [2,3]. Furthermore, Benson and co-workers [4] proposed in 2000 that QDs can emit polarization entangled photon pairs during the radiative decay of the biexcition-exciton (XX-X) cascade:

$$\Psi =\frac{1}{\sqrt{2}}\left(H_{\mathrm{XX}}H_{X}+V_{\mathrm{XX}}V_{X}\right),$$

where H and V are two different polarization states. This feature can be used in the field of quantum cryptography and in advanced quantum optics experiments as quantum teleportation. QDs have the advantage, compared to other sources of entangled photon pairs, that the photons can be emitted on demand, i.e., by using optical or electrical trigger pulses [5,6]. However, these experimental achievements hide the difficulties connected with the experiments. If the QD does not have a certain spatial symmetry (*D*_2d_, or higher), the intermediate X-state is split by the so-called fine structure splitting (FSS), leading to a difference in energy of the photons originating from the X (and also from the XX) decay [4,7,8]. This energy splitting prevents entanglement, since it makes the two decay paths distinguishable, unless the FSS is tuned to the values of the order of the radiative linewidth [9].

**Figure 1:**
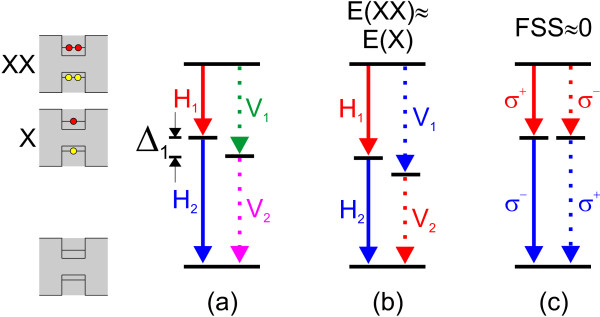
**Sketches of different cascades of the biexciton-exciton decay.** The sketches on the left side show the different states involved in the XX-X decay: biexciton (top panel), exciton (middle panel), and the QD-ground state (bottom panel). **(a)** All four possible transitions have different energies; no entanglement is possible. **(b)** The energies of XX and X are tuned into resonance (same colors-same energy): it is proposed that the time reordering allows restoring entanglement [10]. The transitions of the i-th generation in **(a)** and **(b)** emit linear (*H*_*i*_, *V*_*i*_) polarized light. **(c)** FSS (*Δ*_1_ ≈ 0) tuned to values smaller than the respective linewidth; the so-called which-path information encoded in the FSS is erased, and entanglement is achieved. The transitions in **(c)** emit circularly (*σ*^ + ^, *σ*^−^) polarized light.

Figure 1 shows a sketch of the XX-X decay cascades. The three sketches on the left side show the different states involved in the decay. First the XX decays into X, under emission of a photon, followed by the recombination of the X ending up in the vacuum state. In each of the three situations shown in Figure 1a, 1b, and 1c, the XX can decay via the left (solid line) or right (dashed line) channel under the emission of two photons. The photons emitted by transitions in Figure 1a, b are linearly polarized; in addition, the photons emitted through different channels usually have orthogonal polarization with respect to each other. The transitions labeled with H_i_(V_i_), with *i* = 1, 2, emit horizontally (vertically) polarized photons, defined with respect to a given axis.

In the case of Figure 1a, the energy of the X is split by the so-called FSS, *Δ*_1_; the FSS in (a) is much larger than the linewidth of the emitted lines, leading to energetically well-separated states. The emission energy of the XX is clearly detuned from the one of the X. The different energies (indicated by different colors) of the possible transitions, allow to distinguish the photons originating from the left and the right cascade. This so-called which-path information destroys the polarization entanglement [4,11].

In Figure 1b, the FSS remains unchanged compared to (a), but the average emission energy *E*(XX)of the two perpendicularly polarized XX lines is equal to the one of the X (*E*(X)). This leads within the radiative linewidth to same transition energies of H_1_ and V_2_ as well of V_1_ and H_2_. It has been proposed that the polarization entanglement can be measured when the photons are reordered in time after their emission [10,12-15]. Although, entanglement via the time reordering scheme has not been proven yet, Ding et al. have shown that isotropic biaxial stress can be used to tune the emission energy difference of XX and X to values smaller than the respective linewidth. The observation was explained with a strain-induced tuning of the electron-hole wavefunction overlap [16].

The research presented in this work is motivated by the original proposal from Benson et al. [4], which is equivalent to the XX-X decay configuration depicted in Figure 1c. Here, the emission energies of XX and X are de-tuned with respect to each other, but the FSS is tuned to values smaller than the respective linewidth. In this case, the so-called two first generation transitions (red lines) as well as the two second generation transitions (blue lines) are degenerated in energy. The emitted photons are circularly polarized. Photons from the same generation but from different branches have opposite circular polarization [17]. The spectral overlap removes the which-path information and creates polarization entanglement of the emitted photons [9,11,17,18]. Since the as-grown QDs usually exhibit nonzero FSS, post-growth tuning methods are highly desirable. Several techniques such as thermal annealing [19], lateral [20,21], and vertical electric fields [22], magnetic fields [5], optical Stark effect [9], and anisotropic stress [23,24] can be utilized for this purpose. All the above mentioned techniques have their particular advantages and disadvantages, as will be discussed in this article. This work begins with an introduction on the origin of the FSS, followed by a discussion of the different tuning techniques (some parts are reproduced from the work of Plumhof [25]).

## Excitonic FSS

The different effective masses of heavy holes (HH) and light holes (LH) lead in QDs to different confinement energies of the two upper hole states similar to the confinement of holes in semiconductor quantum wells [26]. This energetic separation makes it more likely for the HH states to be populated, so that one can treat the hole states in a good approximation as pure HH states [8]. The resulting exciton formed by a HH with a spin $m_{J}=|\pm \frac{3}{2}\rangle$ and a conduction band electron with the spin $m_{S}=|\pm \frac{1}{2}\rangle$ can have the following spin states

$$\;M_{d}=|\pm 2\rangle$$

and

$$\;M_{b}=|\pm 1\rangle .$$

Excitons with the spin configuration *M*_*d*_ are commonly known as dark excitons, since optical (single photon) transitions are forbidden due to spin conservation. The excitons with spins *M*_*b*_are called bright excitons, since they can decay under the emission of a circularly polarized photon. This is only the case for bright excitonic states with zero FSS. In the case of non-zero FSS, the bright states are mixed with each other, leading to the emission of linearly polarized light. In the following, the origin of the excitonic FSS and the resulting mixing of the bright excitonic states are discussed.

The origin of the FSS is strongly correlated with the spatial symmetry of the QD, the QDs of symmetry *D*_2d_ or higher show zero FSS. The required spatial symmetry includes the shape of the QD and the symmetry of the underlying crystal. The anisotropy in QDs can have several origins: apart from the QD shape intrinsic strain fields, a crystal of lower symmetry, alloy fluctuations, or alloy ordering can reduce the symmetry [27-31]. For instance, it has been shown that GaAs dots grown on (111) oriented substrates generally exhibit smaller FSS values compared to dots grown on conventional (001) oriented substrates [37,38], in line with theoretical predictions [39]. In addition to symmetry and shape of the QD, also, the volume of the QD modifies the excitonic FSS [32,33]. However, a pure change of the QD-volume will not allow to tune the FSS exactly to zero and will always be related to a potentially unwanted change of the QD emission energy.

The *D*_2d_ symmetry describes a system [34] which is: (1) two-fold rotational symmetric for three different axes (perpendicular to each other); (2) symmetric under reflection at two planes (perpendicular to each other); (3) invariant under a combination of a reflection and a 90 degree rotation around an axis perpendicular to the reflection plane.

**Figure 2:**
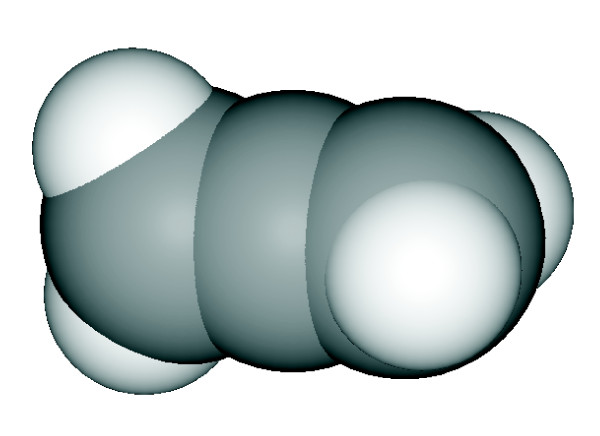
**Structure of an Allene molecule (C**_**3**_**H**_**4**_**) following the*D***_**2d**_**symmetry.** Figure taken from: [35].

In Figure 2, the structure of an Allene molecule, which follows the *D*_2d_ symmetry is presented to illustrate the required symmetry. Due to the random nature of the growth process, as-grown QDs usually do not show this symmetry.

The QD anisotropy mixes the circularly polarized bright excitonic states (*M*_*b*_ = | ± 1〉) and leads to the formation of new bright excitonic states:

(1) $$\frac{1}{\sqrt{2}}(|+1\rangle \pm |-1\rangle ).$$

The photons which are emitted by the recombination of these mixed excitons are linearly polarized, and they have orthogonal polarization with respect to each other. More importantly, the two excitonic states have different energies due to the exchange interaction. The difference in energy is the so-called FSS. The larger the QD-anisotropy, the stronger the coupling of the excitonic states becomes and the larger the magnitude of the resulting FSS. A more rigorous discussion of the origin of the FSS is presented in the work of Bayer et al. [8], as discussed in the following. The Hamiltonian for the electron-hole exchange interaction is given in the basis of the excitonic spin states (| + 1〉,| − 1〉,| + 2〉,| − 2〉) by:

(2) $$H_{\text{ex}}=\frac{1}{2}\left(\begin{array}{c} +\Delta_{0}\;\;+\Delta_{1}\;\;0\;\;\;\;\;0\\ +\Delta_{1}\;\;+\Delta_{0}\;\;0\;\;\;\;\;0\\ \;\;\;0\;\;\;\;\;0\;-\Delta_{0}\;\;\;+\Delta_{2}\\ \;\;\;0\;\;\;\;\;0\;+\Delta_{2}\;\;\;-\Delta_{0} \end{array}\;,\;\right),$$

where: *Δ*_0_ = 1.5(*a*_*z*_ + 2.25*b*_*z*_), *Δ*_2_ = 0.75(*b*_*x*_ + *b*_*y*_), and *Δ*_1_ = 0.75(*b*_*x*_ − *b*_*y*_). The latter term is equivalent to the FSS. The constants describing the spin-spin coupling are given by *a*_*i*_, and *b*_*i*_, with *i* = *xyz*. If the structure exhibits a *D*_2d_ symmetry, *b*_*x*_ and *b*_*y*_ are identical so that the FSS *Δ*_1_ is zero. In this case, the two bright excitons have eigenstates | + 1〉and | − 1〉, which are degenerated and both have the energy $+\frac{1}{2}\Delta_{0}$. The energy splitting of the dark excitonic states, which is given by *Δ*_2_ is always nonzero. The corresponding eigenstates are $\frac{1}{\sqrt{2}}(|+2\rangle \pm |-2\rangle )$, with relative energies $-\frac{1}{2}(\Delta_{0}\pm \Delta_{2})$.

If the symmetry is lower than *D*_2d_, i.e., *b*_*x*_≠*b*_*y*_, the | ± 1〉states are not anymore the eigenstates of the Hamiltonian. In this case, the eigenstates are given by $\frac{1}{\sqrt{2}}(|+1\rangle \pm |-1\rangle )$, and the degeneracy of the energy is lifted. The energy is then given by $\frac{1}{2}(\Delta_{0}\pm \Delta_{1})$, and the difference of the two energies is the FSS (*Δ*_1_). Due to the block diagonal form of the Hamiltonian, the mixing between bright and dark excitons does not occur in this simplified model. On the other hand, if the hole states are not pure but a mixture of HH and LH, also, the LH contributes to the FSS [36].

The exchange interaction can be split into two parts: the so-called short-range interaction (SR) part, arising from the crystal symmetry, i.e., the interaction of electron and hole in the same Wigner-Seitz unit cell, and the long-range interaction (LR) part, when both carriers are in different unit cells, originating from the macroscopic anisotropy of the QD. The LR interaction has two effects: (i) it contributes to the splitting of the bright and dark excitons and (ii) it contributes to the FSS in QDs with shapes of symmetry lower than *D*_2d_. The LR interaction does not contribute to the splitting of the dark excitons. The main effect of the SR interaction is the splitting of the bright and dark excitons, but it also contributes to the FSS.

Even using optimized growth conditions [32,37], the stochastic processes governing the QD formation will always lead to FSS values which are on average too large to satisfy the stringent requirements for entangled-photon generation. Post-growth tuning techniques are therefore essential to increase the yield of QD-based devices capable of generating entangled photon pairs.

## Tuning of the FSS

**Figure 3:**
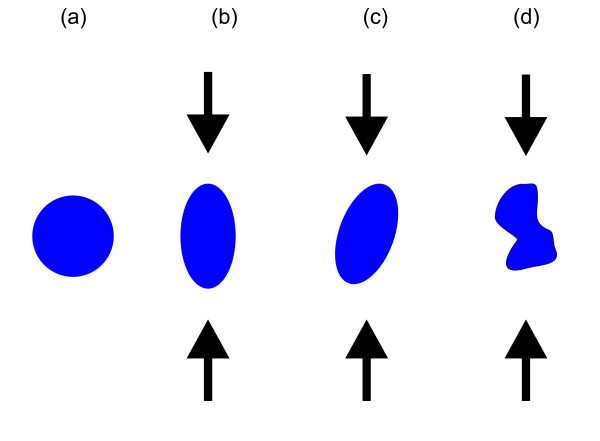
**Cross sections of four different QDs.(a)** Circularly shaped cross section, considered to have a perfect symmetry, i.e., zero FSS. **(b)** Elliptically shaped cross section. **(c)** Same as presented in **(b)**, but with a tilted orientation direction. **(d)** Cross section of arbitrary shape. The black arrows in **(b), (c)** and **(d)** indicate the direction of a unidirectional tuning technique.

Before presenting some experimental works on the tuning of the FSS, we will focus on the theoretical work presented by Singh et al. [40]. In this work, the influence of uniaxial stress on the excitonic FSS in InGaAs/GaAs and InAs/GaAs QDs is discussed by using million atom empirical pseudopotential calculations [41]. As already discussed above, a QD of zero FSS requires a certain (*D*_2d_) symmetry. To simplify the problem, Figure 3 presents a qualitative picture, which reduces the problem to a simple 2D geometrical problem: examples of four QDs of different cross sections/orientations are presented. In Figure 3a, a rotational symmetric QD-cross section is shown, which is considered in this simple model as a QD of ideal symmetry, i.e., with zero FSS. In Figure 3b, an elliptically shaped QD-cross section is illustrated. It is evident that a unidirectional tuning technique, acting along the main elongation axis (indicated by the black arrows), can deform this shape to a circular one, i.e., one expects to be able to tune the FSS to zero. Figure 3c shows a QD of the same cross section as presented in Figure 3b, but now, the elongation axis of the QD is tilted by 20° with respect to the tuning-direction. In this case, a unidirectional tuning technique acting along the vertical axis is not expected to be able to restore the circularly shaped cross section, i.e., the FSS will be nonzero for all tuning magnitudes. In Figure 3d, a QD of arbitrarily shaped cross section is shown. In contrast to the case in Figure 3c, it is evident that in the latter case, no direction exists along which a unidirectional tuning technique would allow deforming the shape to a circle. In this case, we expect the FSS to remain always nonzero. However, in reality, the situation is more complex, i.e., in addition to the shape, also the composition profile, the symmetry/orientation of the underlying crystal, intrinsic strain, and possible consequent piezoelectric fields contribute to the anisotropy and to the FSS [28,33].

**Figure 4:**
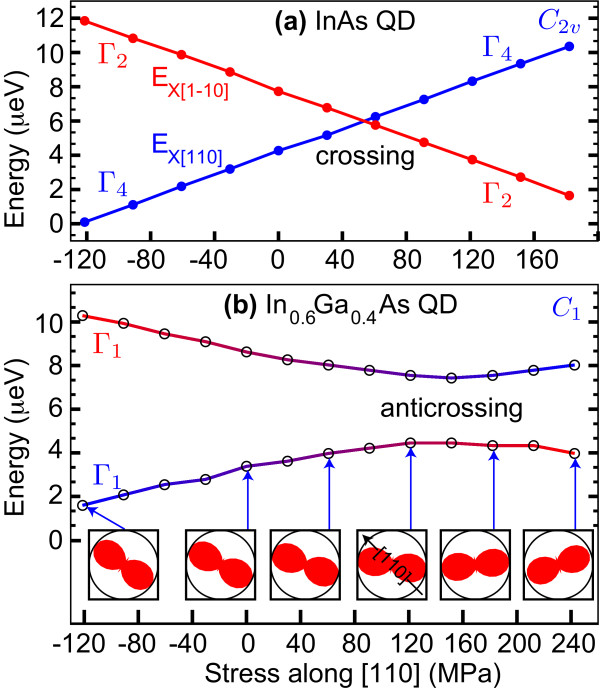
**Theoretical investigations by Singh et al.**[40]** on the influence of uniaxial stress on the FSS.(a)** Energy of the two bright excitons confined in lens-shaped InAs QDs vs. applied stress; the states show a crossing, i.e., zero FSS can be reached. **(b)** Energy of bright excitons confined in a lens-shaped InGaAs QD vs. applied stress; both states show an anticrossing. The insets show how the orientation of the linear polarization is changed if the magnitude of the stress along the [110]-direction is varied. Reprinted figure with permission from R. Singh and G. Bester, *Lower Bound for the Excitonic Fine Structure Splitting in Self-Assembled Quantum Dots*, Phys. Rev. Lett. **104**, 196803, (2010). Copyright 2010, American Institute of Physics.

Figure 4 shows the calculated influence of uniaxial stress on the energy of bright excitons confined in lens-shaped QDs with an elliptical base. The QDs are elongated along the [110]-direction, parallel to the applied uniaxial stress. The results presented in Figure 4a are based on a pure (i.e., non-alloyed) InAs/GaAs QD. The bright excitonic states show a clear crossing, which means that the FSS can be tuned through zero. In contrast, the results presented in Figure 4b are based on an alloyed InGaAs/GaAs QD, showing an anticrossing of the bright excitonic states as the stress is varied, i.e., the FSS remains always nonzero. In addition to the variation of the FSS, a rotation of the linear polarization of the light emitted by the neutral exciton was predicted. The situation in the latter case can be compared with the case illustrated in Figure 3d, where the symmetry is destroyed by the randomness of the QD-shape, whereas in the case of Figure 4b, the randomness of the alloy is responsible for the lowered symmetry.

Similar results, based also on atomistic theory, were obtained by Bryant et al. [42]. They studied the influence of stress on InAs/GaAs QDs embedded in a bendable GaAs bridge. They explain the rotation of the linear polarization by a change of the phase between the mixed bright excitonic states. Recently, the relation between magnitude of the FSS and polarization angle has been described more in detail in the work of Gong et al. [43].

All these theoretical works predict a non vanishing FSS for QDs which do not exhibit a certain symmetry. However, to create polarization entangled photon pairs zero FSS is not strictly required, as it is sufficient to tune the FSS down to values of several μeV[44], which means that also the FSS of QDs of a lower symmetry can be tuned to values small enough to create polarization entangled photon pairs.

## Tuning of FSS by annealing

Thermal annealing of the QD structures allows the FSS to be modified irreversibly [19,45]. This technique has been mainly employed on InGaAs/GaAs QDs and is based on the diffusion of In in InGaAs/GaAs QDs. Annealing of the QDs at temperatures above about 700°C leads to a net in-diffusion from the in-richer center of the QD to the in-poorer surrounding of the QD. To control this process, one can either set a fixed temperature and vary the duration of the annealing process [45], or one can keep the annealing time fixed and vary the temperature [19]. The change of the QD-composition profile leads to a change of the QD-extension and to a modification of the effective shape of the confinement potential.

**Figure 5:**
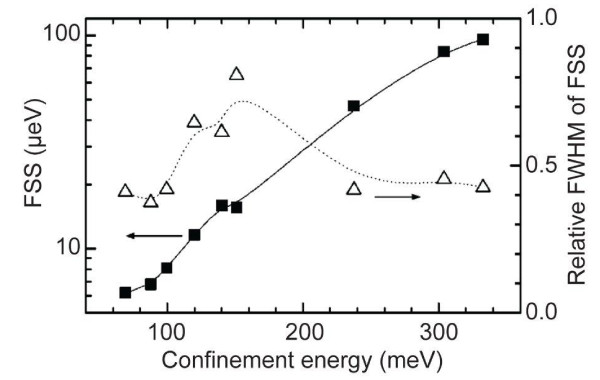
**Results from Langbein et al.**[19]. Average FSS (solid squares) and the relative FWHM of the corresponding FSS distribution (open triangles) vs. the confinement energy. Reprinted with permission from Langbein et al., *Control of fine-structure splitting and biexciton binding inIn*_x_*Ga*_1-x_*As quantum dots by annealing.*, Phys. Rev. B **69**, 161301(R), (2004). Copyright 2004, American Institute of Physics.

Figure 5 shows the results from the work of Langbein et al. [19]. It shows the average FSS (solid squares) and the relative full width at half maximum (FWHM) for each of the measured distributions (open triangles) vs. the confinement energy, which is defined by the QD ground state energy with respect to the energy of the wetting layer. The recorded data are measured on samples being annealed for 30 s at different temperatures (800 to 960°C). The confinement energy changes from 332 meV for the not annealed sample to 69 meV for the sample which was annealed at 960°C. At the same time, the FSS decreases from 96 down to 6 μ*eV*. The relative FWHM of the distributions does not change remarkably (see open triangles), showing that the FSS of all QDs of the ensemble are tuned in a similar way.

The evolution of the FSS of single dots upon annealing has been studied by Ellis et al. [46] and by Seguin et al. [47]. The advantage of annealing as tuning technique is that the tuning is permanent.

## Tuning of FSS by magnetic fields

A very powerful technique to tune the FSS is represented by magnetic fields, where one generally distinguishes between two configurations: (i) Faraday configuration, i.e., with the magnetic field oriented along the growth direction [8], (ii) Voigt configuration, i.e., with the magnetic field aligned along an axis perpendicular to the growth direction [8,48]. In the Faraday configuration, the magnetic field lifts the degeneracy of the excitonic states of positive and negative spin projections, but it does not allow to tune the FSS to zero [48]. In the Voigt configuration, the in-plane magnetic field affects the lateral symmetry of the QDs and, thus, also the FSS. Additionally, it couples bright and dark excitonic states, *M*_*b*_and *M*_*d*_, making the dark states bright [48-50].

**Figure 6:**
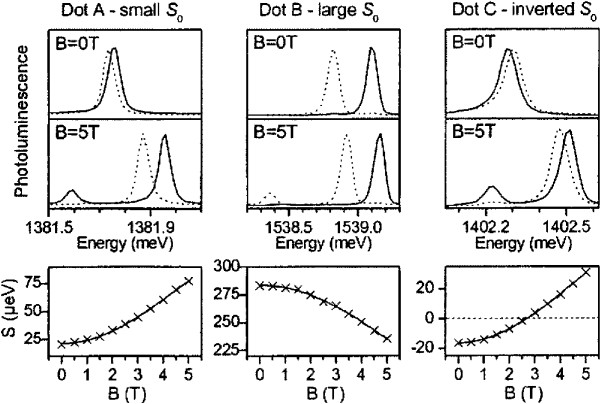
**Results from Stevenson et al.**[48]**obtained by application of an in-plane magnetic field.** Three different QDs A, B, and C having at zero magnetic field a FSS of 22,284, and −16 *μeV*, respectively. The top (middle) panels show spectra of the X-emission at a magnetic field of *B* = 0 T (*B* = 5 T). The dashed and solid lines stand for the spectra recorded at perpendicular polarization angles, respectively. The bottom panels show the behavior of the FSS (S) as it is tuned by the magnetic fields. Reprinted with permission from Stevenson et al., *Magnetic-field-induced reduction of the exciton polarization splitting in InAs quantum dots*, Phys. Rev. B **73**, 033306, (2006). Copyright 2006, American Institute of Physics.

In Figure 6, the results from reference [48] are shown. The behavior of the emission of Xs confined in three different InAs QDs (A, B, and C) under an in-plane magnetic field is presented. The upper panels show the QD spectra at zero magnetic field, where the two displayed lines (dashed/solid) represent the spectra taken for two perpendicular polarization directions. It can be seen that the different QDs have different FSS at *B* = 0 T. QD A has a small FSS of only *S*_0_ = 22 μ*eV*, QD B has a comparably large splitting of *S*_0_ = 284 μ*eV*, and QD C has a negative splitting of *S*_0_ = −16 μ*eV*. The reason for the negative sign of the FSS in the case of QD C is that the higher energy emission line is polarized perpendicularly to the one of QDs A and B (compare upper three panels). Other works consider only positive values of the FSS and define an angle for the polarization orientation [24,51]. In the middle panels, the excitonic spectra for the same QDs are presented as an in-plane magnetic field of 5 T is applied. It can be seen that the distance between the two main peaks, i.e., the FSS has changed, and a third line appeared at lower emission energies, which is ascribed to transitions of the former dark excitonic states. In the bottom panels, the FSS (S) is plotted vs. the magnetic field. It can be seen that the FSS of QD A increases from 22 to 77 μ*eV*as B is tuned from 0 to 5 T. In the case of QD B, the FSS decreases from 284 to 235 μ*eV* as B is varied. The right bottom panel presents the tuning behavior of QD C. In this case, the FSS can be tuned from −16 through 0 to +31 μ*eV*. The different tuning behaviors are attributed to different initial FSSs and to different g-factors of the particular QDs. It is shown by the same authors, that this technique is able to tune the magnitude of the FSS to values small enough to achieve polarization entanglement [5]. The disadvantage of the tuning by magnetic fields is that fields of several Tesla have to be applied in order to reach reasonably small FSSs. This hinders an on-chip integration of the tuning technique and makes it less appealing for real applications.

## Tuning of FSS by the optical Stark effect

**Figure 7:**
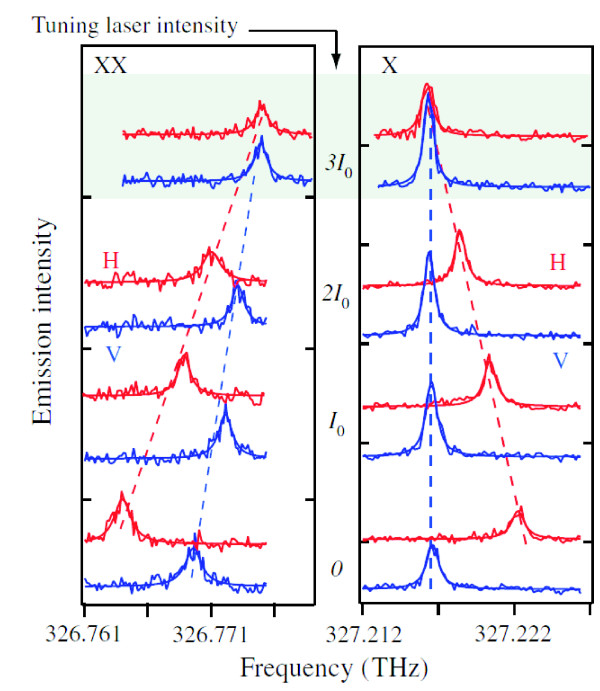
**Results from Muller et al.**[9]. Spectra of the biexcitonic (left) and of the excitonic (right) transitions measured for vertical (V) and H polarization (H) for different tuning laser intensities *I*; where an intensity of *I*_0_corresponds to a Rabi frequency of *Ω*/2*Π* = 8.3 GHz. Reprinted with permission from Muller et al., *Creating Polarization- Entangled Photon Pairs from a Semiconductor Quantum Dot Using the Optical Stark Effect*, Phys. Rev. Lett. **103**, 217402, (2009). Copyright 2009, American Institute of Physics.

Another way to tune the FSS is to use the optical Stark effect as presented in the work of Muller et al. [9]. In this case, the electric field of a continuous-wave laser is used to tune the FSS. A laser, de-tuned by 25.5 GHz from the XX transition, is coupled into an in-plane waveguide containing the QDs. The interaction of the QD with the light-field modifies the QD emission. The optical Stark effect increases with the laser intensity and decreases with the de-tuning of the laser frequency, with respect to the investigated transitions [52]. Figure 7 shows how the frequency of the exciton and biexciton emission changes as the intensity of the tuning laser is increased, where an intensity of *I*_0_ corresponds to a Rabi frequency of *Ω*/2*Π* = 8.3 GHz. In the left panel, the spectra of the two (H, V) polarized fine structure split biexcitonic emission lines are presented. Both emission lines are tuned in energy and come closer as the intensity of the tuning laser is increased. Finally they merge at *I* = 3*I*_0_.

In the right panel of Figure 7, the same is shown for the excitonic spectrum. Again, both the emission lines get closer as *I* is increased, and they finally overlap at an intensity of *I* = 3*I*_0_. Interestingly, the V-polarized excitonic emission is only slightly effected by *I*, which can simply be explained by a coupling of the linearly polarized laser field with mainly the H-polarized excitons. It is shown that QDs with FSSs of more than 20 μ*eV* can be tuned to values small enough to create polarization entangled photon pairs. The presented technique has the advantage that one can precisely *in-situ* modify the FSS, but on the other hand, an additional laser source is needed, making the on chip implementation more complicated.

## Tuning of FSS by the quantum-confined stark Stark effect

Another promising approach is the so-called quantum-confined Stark effect, where static electric fields are utilized. The electric field can have different configurations, i.e., it can be applied either vertically (i.e., parallel to the growth direction) [22] or laterally (i.e., perpendicular to the growth direction) [20,21,53]. Gerardot et al. [21] investigated the influence of a lateral electric field on the FSS of InGaAs/GaAs QDs. They placed two Schottky contacts separated by 15 *μ*m on the surface of the sample in order to apply an in-plane electric field to the QDs, being located 130 nm below the surface.

**Figure 8:**
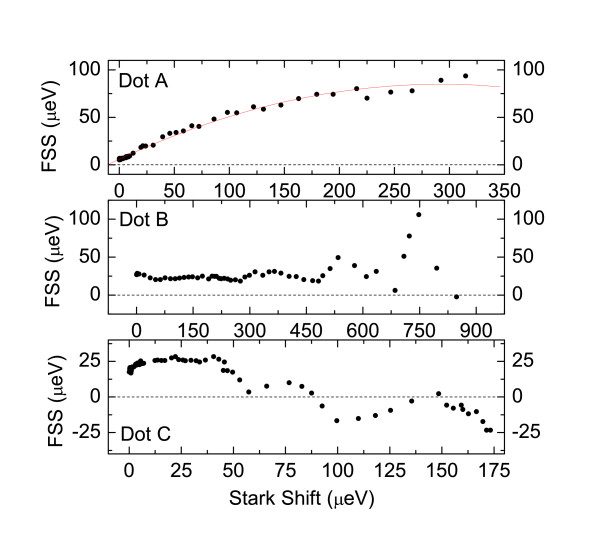
**Results from the work of Gerardot et al.**[21]. FSS of three different InGaAs/GaAs QDs (**A, B** and **C**) vs. their emission energy, as a lateral electric field is applied. Reprinted with permission from Gerardot et al., *Manipulating exciton fine structure in quantum dots with a lateral electric field*, Appl. Phys. Lett. **90**, 041101, (2007). Copyright 2007, American Institute of Physics.

In Figure 8, the relation of FSS and emission energy for three different QDs is presented, as the lateral electric field is varied. All QDs show different behaviors: the emission energies can be tuned by values of up to 1 meV, whereas the FSS varies in a non trivial way. In the case of QD A, the FSS shows a square root-like dependence with the applied field, and it can be tuned in a range of around 90 μ*eV*. For QD B and C, the FSS shows an oscillatory behavior. In QD B, the FSS only approaches zero, whereas in case of QD C, the FSS crosses zero.

The three arrows in the bottom panel depict measurements, where the polarization orientation of the emission was characterized. No rotation of the polarization was reported. The main drawback of the measurements presented in this work is that the application of a lateral electric field leads to a separation electrons and holes, which leads to a decrease of the emission intensity, making together with an increase of the emission linewidth this technique less appealing for future applications. Similar observations were reported in the work of Vogel et al. and Kowalik et al. [20,53]. Another drawback of this method is that the roughness of the the electric contacts does not allow for a precise alignment of the electric field.

**Figure 9:**
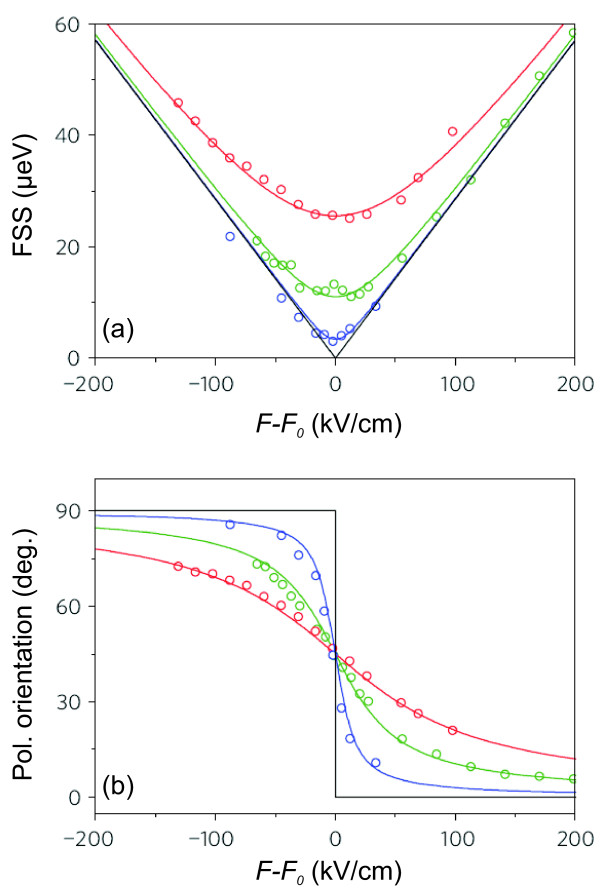
**Results from the work of Bennett et al. describing the influence of a vertical electric field on the emission of InAs/GaAs QDs**[22]**.(a)** FSS of several QDs vs. the relative electric field *F* − *F*_0_, where *F*_0_is the field where the FSS of each QD reaches its minimum, *s*_0_. **(b)** Orientation of the linear polarization of the emitted light with respect to the crystal axes vs. the relative electric field for the same QDs as presented in **(a)**. Reprinted by permission from Macmillan Publishers Ltd: Nature Physics, Bennett *et al.*, *Electric-field-induced coherent coupling of the exciton states in a single quantum dot*, Nature Physics **6**, 947, (2010), copyright 2010.

An elegant way to overcome those problems is to use vertical electric fields, as presented in the work of Bennett et al. [22]. They designed a semiconductor heterostructure, which reduces the carrier tunneling rate. This allows for the application of high-electric fields to high-quality InAs/GaAs QDs without a noteworthy loss of emission intensity. Their results are presented in Figure 9. Figure 9a shows how the FSS changes as the relative electric field *F* − *F*_0_ is varied, where *F*_0_ is the value of the electric field for which the FSS reaches its minimum magnitude, *s*_0_. For FSS≫ *s*_0_, an almost linear relation between relative electric field *F* − *F*_0_ and FSS was reported. Due to the different center of masses of the electron and hole wave functions, the excitons confined in QDs usually have a permanent dipole moment *p* = *e*·*d*, where *e* is the value of the elementary charge, and *d* is the distance between the center of masses of electron and hole wave functions [54]. When an electric field *F* is applied to the QD, the energies *E* of the two excitonic lines composing the bright exciton behave according to:

$$E=E_{0}-\mathrm{pF}+\beta F^{2},$$

where *E*_0_ is the energy of the Xs at *F* = 0 kV/cm, and *β* stands for its polarizability. For large FSS, the two excitonic states can be imagined as radiating electric dipoles, being aligned along the [110]/[1$\bar{1}$0] directions of the crystal. The alignment of the excitonic dipoles is transferred to the orientation of the linear polarization of the light emitted by the recombination of the excitons. Since the polarizability does not depend on the in-plane anisotropy of the QD, it does not contribute to the relative change of the two excitonic energies (i.e., to the FSS) [55]. Due to the different confinement potentials of the excitons along the two directions, their permanent dipole moments *p*_1_ and *p*_2_ exhibit different values [54], leading to a linear change of the FSS:

$$\Delta \mathrm{FSS}=(p_{1}-p_{2})\mathrm{F.}$$

This relation explains why the FSS changes linearly with *F* for FSS > > *s*_0_. In the region close to *F* − *F*_0_ = 0 kV/cm, this simple approximation is not valid. The fact that for FSS > > *s*_0_, all QDs show a similar relative change of the FSS as *F* is varied indicates that all QDs have similar in-plane anisotropy. In Figure 9b, the orientation angle of the polarization of the two excitonic emission lines with respect to the crystal axes [110]/[1$\bar{1}$0] for three different QDs (same as in Figure 9a) is shown. It can be seen that the polarization rotates faster, the smaller is the minimum value of the FSS, and that the polarization rotation mainly takes place in the region of the lowest FSS magnitudes. The joint effect of changing polarization orientation and modification of the FSS can be explained by the afore mentioned anticrossing of the bright excitonic states. Despite the clear anticrossings, it has been demonstrated that this tuning technique can be used to tune the FSS of some QDs to values small enough for the creation of entangled photon pairs.

## Tuning of FSS by anisotropic stress

**Figure 10:**
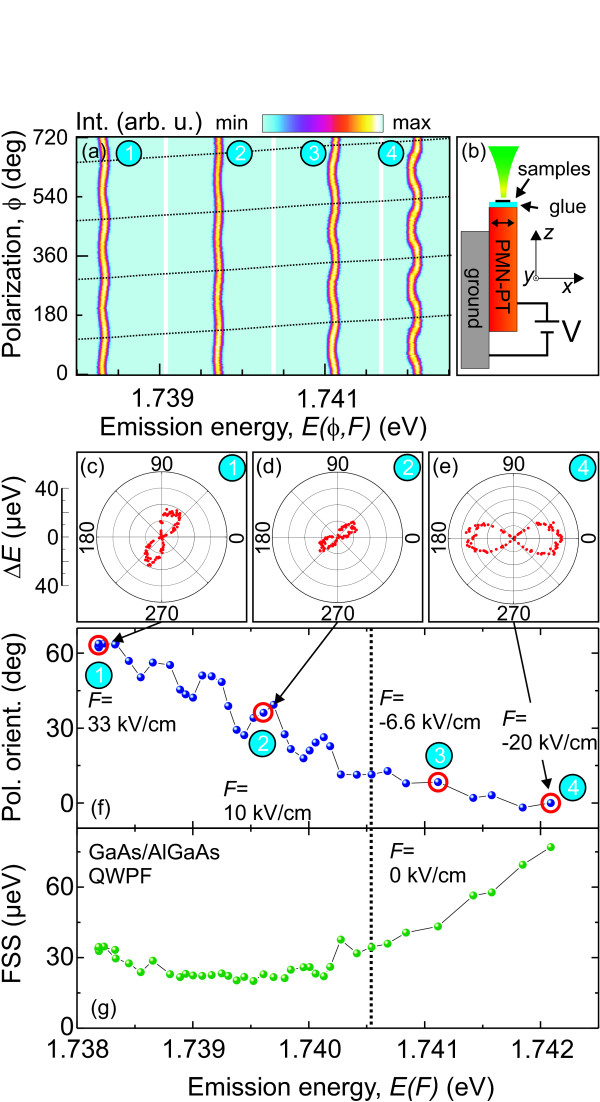
**Results from the work of Plumhof et al.**[24]**.** Behavior of the X-emission of a GaAs/AlGaAs QWPF as anisotropic biaxial stress is applied. **(a)** Color-coded PL intensity vs. polarization angle, *ϕ*, and emission energy, *E*(*ϕF*), for different values of *F* applied to the PMN-PT (the field values for the panels 1 to 4 are indicated in **(f)**). The intensity is normalized to the highest intensity value for each polarization measurement (1-4). The dashed lines indicate the rotation of the polarization orientation. The *x* direction in **(b)** is parallel to *F* and to *ε*_∥_and corresponds to polarization angles of 0°, 180°,.. and coincides with the polarization orientation of the high-energy component at *F* = −20 kV/cm (panel 4). **(b)** Sketch of the device consisting of a QD-membrane glued on a side of a PMN-PT crystal. **(c)(e)** Polarization dependence in polar coordinates of the relative emission energy, *ΔE*, (i.e., the mean emission energy, *E(F)*, for each value of *F* is subtracted) for panels 1, 2, and 4, respectively, of **(a)**. **(f)** Polarization orientation of the high-energy component with respect to *x* vs. *E(F)*. The dots marked by red circles correspond to the data shown in **(a)**. **(g)** FSS vs. *E(F)*. Reprinted figure with permission from Plumhof *et al.*, *Strain-induced anticrossing of bright exciton levels in single self-assembled GaAs/Al*_x_Ga_1-x_ As and In_x_Ga_1-x_*As/GaAs quantum dots*, Phys. Rev. B. **83**, 121302(R), (2011). Copyright 2011, American Institute of Physics.

The last tuning technique presented in this work is based on anisotropic stress. More details can be found in the work of Plumhof et al. [24]. In order to apply stress to different kinds of QDs, 150-200 nm thick membranes containing QDs were created by selective etching techniques [56] and integrated on a piezoelectric crystal (PMN-PT) [57] which allows anisotropic stresses to be applied to the QDs. The anisotropic stress created by the PMN-PT is composed by a component, *ε*_∥_, parallel to the electric field, *F*, applied to the PMN-PT and by a component, *ε*_⊥_, perpendicular to *F*, with the relation *ε*_⊥_ = −0.7 × *ε*_∥_. Figure 10b shows a sketch of the device, where the electric field is applied along the *x* direction, which is parallel to *ε*_∥_. The [001]-direction of the membranes is pointing along the *z*-direction. The [1$\bar{1}$0] direction of the membrane is tilted by an angle of approximately 20° with respect to the *x* axis.

In Figure 10, the behavior of the emission of an X confined in a GaAs/AlGaAs quantum well potential fluctuation (QWPF) is presented as anisotropic stress is applied to the QD-membrane. (A QWPF is a local variation of the quantum well, acting as a comparably shallow confinement potential [7,29]).

Figure 10a shows the polarization resolved color-coded PL-intensity map of the X-emission as a function of the emission energy for different electric fields *F* =33, 10, −6.6, −20 kV/cm (labeled in the figure by the numbers 1 to 4) applied to the PMN-PT. The intensities of each line (1-4) are normalized to their maximum. Three effects are mainly observed in (a): (i) The emission energy *E*(*F*,*ϕ*) shifts by approximately 4 meV. (ii) The polarization orientation of the excitonic emission, which is related to the phase of the wavy pattern, rotates by more than 60° (indicated by the dashed lines). (iii) The magnitude of the FSS, i.e., the amplitude of the oscillations of the wavy patterns first decreases (points 1→2) and then increases again (2→4).

In the following, the polarization angles are given by the orientation of the high-energy emission line of an exciton with respect to the direction of *F*. In Figure 10c,e, the polarization dependence of the relative peak positions *ΔE* = |*E*(*ϕ*,*F*) − *E*(*F*)| for points 1, 2, and 4 is illustrated in polar coordinates, where *E*(*F*)is the average emission energy for a certain value of *F*. They show clearly that FSS and polarization orientation change for different fields *F*. Figure 10f displays the polarization orientation vs. *E*(*F*), as *F* is varied from *F* = 33 to −20 kV/cm. Figure 10g shows the magnitude of the FSS vs. *E*(*F*). Going from low to high emission energies, i.e., from tensile to compressive strain, the FSS first decreases down to a minimum value of about 20 μ*eV*and then increases again. Simultaneously, the polarization orientation rotates by around 70° (see Figure 10f). The origin of the oscillations of the polarization in Figure 10f is ascribed to the defects in the QWPF surrounding, which act as traps for charge carriers. The presented data describe a clear anticrossing of the bright excitonic states. In the same work, it is also shown that anisotropic stress can be used to tune the FSS of different kinds of semiconductor QDs by several tens of μ*eV*, down to values, which are expected to be small enough to create polarization entangled photon pairs.

Strain-dependent calculations based on *k*·*p*and configuration interaction models show that the minimum reachable FSS highly depends on the alignment of stress with respect to the QD elongation direction (compare also with Figure 3b,c). Furthermore, it is demonstrated that the anisotropic stress produces a rotation of the hole wave function [24]. The calculations show that the reason for the tuning of the FSS is not the physical deformation (which is only a few per mill) but the influence of the stress on the band structure. The stress leads to a mixing of heavy with light hole band and to an anisotropy of the effective mass, which finally modify the FSS. These results demonstrate that stress is a powerful tuning technique.

The advantage of stress is that it can easily be combined with other tuning techniques, like electric [58] or magnetic fields which provides another degree of freedom. An on-chip combination of QDs embedded in a light emitting diode and stress would allow to electrically trigger the QD emission and to tune the FSS by using anisotropic stress. The disadvantages of stress as a tuning technique are that the piezoelectric materials have to be actively stabilized in order to avoid creeping of the piezo, and it is difficult to apply the stress only locally and stress, as demonstrated after submission of this manuscript by Trotta et al. [59].

## Conclusions

Several methods are presented, which allow the excitonic FSS of semiconductor QDs to be tuned after growth. All the presented tuning techniques like annealing, magnetic fields, optical Stark effect, electric fields, and stress are able to tune the FSS of particular QDs to magnitudes, which are sufficiently small to create polarization entangled photon pairs. All the presented methods have their particular advantages and disadvantages. Magnetic fields for example require cumbersome experimental setups, which makes an on-chip integration difficult, annealing does not lend itself to *in-situ* monitoring, but it has the advantage that the FSS can be permanently tuned to low magnitudes. Vertical electric fields are proven to be an effective tuning method, but the use of static electric fields for tuning makes it difficult to electrically trigger the QD emission. A problem which all the techniques presented so far share is that they are not able to tune the FSS of all the QDs to sufficiently small magnitudes. This problem could be solved by combining two or more tuning techniques, like static electric fields, optical stark effect, and stress.

## Competing interests

The authors declare that they have no competing interests.

## Authors’ contributions

The manuscript was written by JDP with help from RT; the work was supervised by AR and directed by OGS. All authors read and approved the final manuscript.
